# Sampling the fish gill microbiome: a comparison of tissue biopsies and swabs

**DOI:** 10.1186/s12866-021-02374-0

**Published:** 2021-11-10

**Authors:** Morag Clinton, Adam J. Wyness, Samuel A. M. Martin, Andrew S. Brierley, David E. K. Ferrier

**Affiliations:** 1 Scottish Oceans Institute, Gatty Marine Laboratory, School of Biology, University of St Andrews, St Andrews, Fife, KY16 8LB UK; 2 Department of Veterinary Medicine, University of Alaska Fairbanks, Fairbanks, AK 99775 USA; 3 Coastal Research Group, Department of Zoology and Entomology, Rhodes University, Makhanda (Grahamstown), 6139 South Africa; 4 School of Biological Sciences, University of Aberdeen, Aberdeen, AB24 2TZ UK

**Keywords:** Experimental design, Sampling methodology, Gill microbiota, Microbial assemblages, Aquaculture, Atlantic salmon, Veterinary microbiology

## Abstract

**Background:**

Understanding the influence of methodology on results is an essential consideration in experimental design. In the expanding field of fish microbiology, many best practices and targeted techniques remain to be refined. This study aimed to compare microbial assemblages obtained from Atlantic salmon (*Salmo salar)* gills by swabbing versus biopsy excision. Results demonstrate the variation introduced by altered sampling strategies and enhance the available knowledge of the fish gill microbiome.

**Results:**

The microbiome was sampled using swabs and biopsies from fish gills, with identical treatment of samples for 16S next generation Illumina sequencing. Results show a clear divergence in microbial communities obtained through the different sampling strategies, with swabbing consistently isolating a more diverse microbial consortia, and suffering less from the technical issue of host DNA contamination associated with biopsy use. Sequencing results from biopsy-derived extractions, however, hint at the potential for more cryptic localisation of some community members.

**Conclusions:**

Overall, results demonstrate a divergence in the obtained microbial community when different sampling methodology is used. Swabbing appears a superior method for sampling the microbiota of mucosal surfaces for broad ecological research in fish, whilst biopsies might be best applied in exploration of communities beyond the reach of swabs, such as sub-surface and intracellular microbes, as well as in pathogen diagnosis. Most studies on the external microbial communities of aquatic organisms utilise swabbing for sample collection, likely due to convenience. Much of the ultrastructure of gill tissue in live fish is, however, potentially inaccessible to swabbing, meaning swabbing might fail to capture the full diversity of gill microbiota. This work therefore also provides valuable insight into partitioning of the gill microbiota, informing varied applications of different sampling methods in experimental design for future research.

**Supplementary Information:**

The online version contains supplementary material available at 10.1186/s12866-021-02374-0.

## Introduction

The microbiome is considered critical to health in many aquatic organisms, which requires maintenance of a diverse microbial community [1]. Study of the microbiome therefore presents an exciting avenue of research in fish. There is a growing body of data on fish microbiomes, examining resident microbiota of different tissues, and how communities respond to changes such as diet and environment. While only a small number of true mutualistic relationships have thus far been described in teleosts [2–6], many researchers propose a role for the resident microbiota of fish in immune function and disease resistance, as well as in nutrient uptake for enhanced growth [7–11]. Under ‘normal’ conditions, the microbiota is in homeostasis, however, whenever this breaks down ‘dysbiosis’ is considered to occur; whereby a disrupted microbiome results as a consequence of, or predisposition to, disease [12–14]. Gill microbiota are hypothesised to assist in prevention of infectious disease through competition and production of community modulatory compounds [15]. Shifts in community structure might therefore predispose the fish to pathology through increased growth or ingress of undesirable microbes that might cause disease, or impair tissue function [16, 17]. An understanding of the microbial ecology of fish tissues is clearly essential in understanding the role bacteria might play in fish health, and for exploring the existence of any mutualistic relationships. Of particular interest to researchers are the relationships between important aquaculture species such as Atlantic salmon (*Salmo salar*) and their microbial consortia [18, 19]. Many critical uncertainties remain as yet in this area of research, particularly regarding the involvement of microbial communities and other factors that influence the complex suite of gill disorders experienced by these fish [20–23].

Gill health in farmed fish is fundamental to overall health and their performance. As such, research is beginning to examine the diversity and structure of the gill-specific microbial communities across a number of fish species [24, 25]. Gills are important immunologically active mucosal tissues [26, 27], considered the site of much pathogen ingress due to their environmental exposure. Recent evidence has shown significant divergence between the microbial community of gills and surrounding water in numerous fish species [28–30]. It is considered that the microbiota of external epithelial tissue, although influenced by external factors [31, 32], is distinct from environmental populations in part due to host factors, as well as existing microbial interactions [19, 33, 34]. Although recent studies have explored the distribution across the gill ultrastructure for specific microbial taxa, such as the pathogen *Yersinia ruckeri* [35], the potential for varied population distribution of microbial assemblages across the topography of gill tissue remains to be resolved. Research on the Atlantic salmon gastrointestinal tract (GIT), however, suggests existence of a ‘core’ microbiota for specific tissue regions; a population of consistently resident microbes suggested to be specialised to specific compartments, with utility in digestion or defence [11, 36, 37]. Such a community remains to be established for external epithelial tissues such as skin and gills.

Development of standard methods for exploration of different facets of the microbiome is essential to ensure representative, reproduceable research with recommendations to avoid bias during the sampling and processing [38–40]. Variations in sampling and storage as well as DNA extraction protocol have all been shown to impact the final outputs of data in the study of terrestrial vertebrates [41–44]. These early stages on the pipeline can have impacts on the high-throughput sequencing results of microbial communities. However, less information is available regarding protocol suitability for the study of aquatic organisms [39, 45, 46]. The existing fish-specific studies testing the impact of storage method [47] and extraction protocols [48] on surveys of community composition report variation in results with altered techniques, but the impact of sampling methodology is as yet poorly understood. Whilst previous research has also demonstrated the importance of sample location in isolation of particular compartment-specific subsets of GIT microbial communities [49, 50], there exists no published data regarding the impact of sampling methodology or localisation on gill communities. Traditional microbiological culture techniques obtain microbiota from tissue sections directly, or by swabbing the gill surface [51], and remain important in pathogen diagnosis. Many marine microbiota are considered particularly difficult to culture however, and sequencing is often more successful in isolation of diverse communities. Existing publications in molecular isolation utilise a variety of sampling techniques from gills including swabbing [24, 52], and biopsies [30, 53], but none so far have contrasted these two methodologies.

Biopsies are more invasive than swabbing, and therefore often less desirable in many contexts, however, their use can be warranted in specific circumstances. In the study of human epithelial tissue for example, although swabbing is considered a suitable proxy and preferable alternative to biopsy excision for isolation of epidermal microbiota in many clinical scenarios [54, 55], research in human health identifies a lack of comparability between swab and biopsy in sampling different human epithelial tissues in a number of instances [56, 57], necessitating the use of biopsies for microbial isolation in some circumstances. Overall though, there remains a lack of consensus within the human literature as to the comparability of these methods in the study of the GIT and healthy skin microbiota [42, 58–60]. Although the methodologies of swabs and biopsies have thus been compared in many different human medical scenarios, this information is lacking in the study of fish. Crucially, no information is available either regarding the distribution of any site-specific microbiota of gill tissue that might inform best-practice in sampling.

Here, we compare the utility of swabbing and biopsy sampling in Atlantic salmon, a commercially important finfish, and demonstrate that the two sampling techniques provide very different profiles of the resident microbiome. Swabbing provides a wider coverage of the diversity of microbes, presumably living on the swab-accessible surfaces of the gill, whereas biopsies provide a more restricted coverage of diversity, but importantly seem to include swab-inaccessible taxa, many of which may be of relevance to gill pathogenesis. These results should inform future experimental design in the study of gill microbiota, as well as provide insight into any anatomical influences on microbial community composition.

## Results

### Community level differences between swab and biopsy-derived samples

**Fig. 1:**
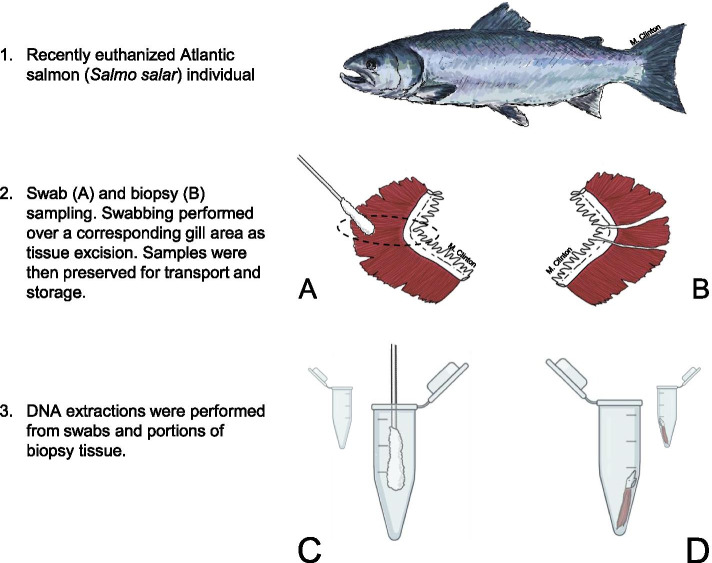
Sampling methodology. Biopsy excision and swab application was performed on the left and right sides of fish respectively. The first gill was sampled in situ for each methodology prior to fixation of samples. Biopsies were excised as full tissue thickness sections including supporting cartilage and filament tissue. Swabbing (**A**) was performed over a corresponding gill region to tissue excision (**B**). Samples were subsequently utilised in DNA extractions (**C, D**). Swabs were scraped using a sterile blade and extractions performed on this dislodged material as well as directly from the swab (**C**). A fraction (10 mg +/− 2 mg) of each biopsy tissue sample was processed for DNA extraction (**D**), with replication achieved through successive extractions from further biopsy sub-samples. This file was partially generated using BioRender

A total of 24 samples were obtained, of swab and biopsy samples from each individual of Atlantic salmon as well as environmental microbial communities. Beyond different collection strategies, sampled material was treated identically to explore the impact of sampling methodology on results (Fig. 1). From swab sampling, following DNA extraction, amplification and sequencing, a total of 119,258 pre-processed reads were obtained, with an average of 11,435 quality-filtered reads per sample after processing. Corresponding biopsy samples resulted in a total of 80,111 pre-processed reads and an average of 2352 quality-filtered reads per sample after processing. High frequency filtered reads (considered those where over 1000 reads where identified) were explored using BlastN and identified to be from salmonid DNA in all cases. Filtration of reads removed a high number of these sequences from biopsy samples in particular (Additional file 1). Rarefaction curves suggest adequate depth of sequencing was achieved based on sequencing plateaus (Additional file 1). From the swab-derived samples a total of 303 Amplicon Sequence Variants (ASVs) were identified. This equated to 260 genera, 164 families, 99 orders, 51 classes and 24 phyla. For biopsy-derived samples, total counts of ASVs at different taxonomic levels from the same individuals were overall lower. A total of 131 ASVs, representing 114 genera, 86 families, 57 orders, 33 classes and 14 phyla were obtained.

**Fig. 2:**
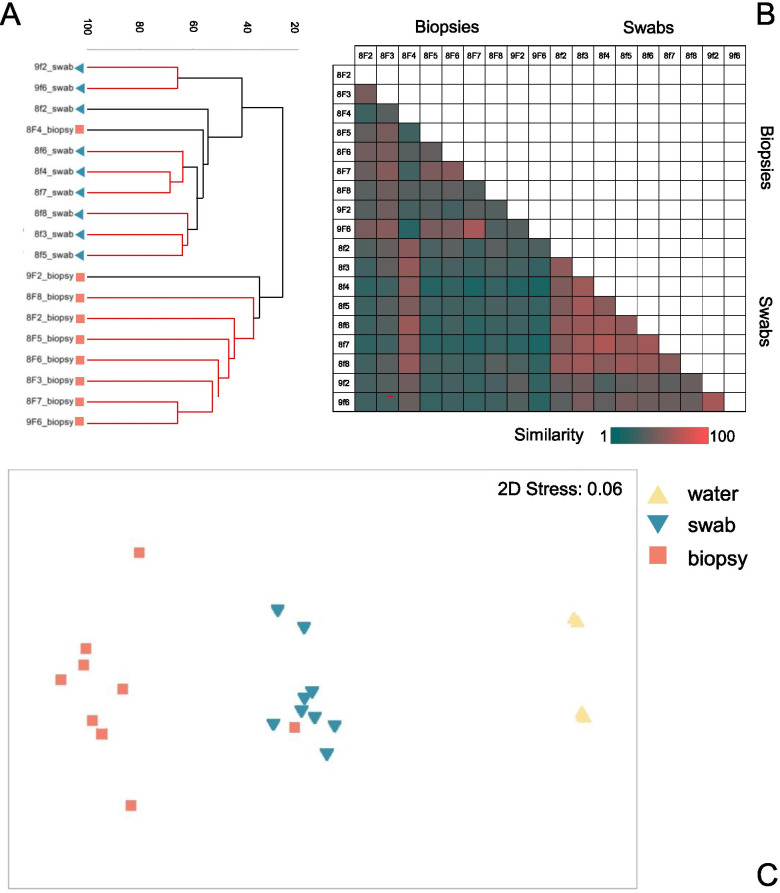
Beta diversity indices of swab and biopsy results. **A**. Hierarchical cluster analysis of square root transformed Bray-Curtis data (shown in panel **B**) demonstrates group average clustering of community results between swab and biopsy samples. Red lines group statistically indistinguishable samples, and black lines indicate statistically significant differences between samples (with significance set at 0.01 and performed using SIMPROF testing in Primer 7). **C**. non-metric multi-dimensional scaling plot of square root transformed Bray-Curtis data illustrates resemblance of microbial communities obtained by swabbing, biopsy excision and from surrounding environmental populations. Generated using Primer 7

Non-metric multi-dimensional scaling (nmMDS) of microbial community based on non-rarefied datasets [61] demonstrates an association of samples by sampling methodology (Fig. 2). Despite being obtained from the same fish, concurrently obtained swabs and biopsies do not show a close association, ordinating principally by method of collection instead. Plotted variables demonstrate no clear association of samples obtained from the same individual. Group average hierarchical cluster analysis of overall microbial community sequencing (using results of standardised Bray-Curtis similarity analysis) supports these results (Fig. 2). Hierarchical clustering demonstrates six statistically distinct clusters, with clusters containing only swab or biopsy samples, never both. Statistically significant clusters can be grouped visually to two main clusters overall (subsequently referred to as Cluster 1 and Cluster 2). Cluster 1 is composed mainly of swab-derived isolates (90% swabs, 10% biopsies), and Cluster 2 is composed entirely of gill biopsy communities (100%). Statistical differences between samples were identified using the inbuilt SIMPROF testing of the Primer 7 cluster function, with significant (*p* = < 0.01) differences highlighted in black, and samples with a lack of significant variation clustered with branches coloured red. Intra-individual differences in results are identified with sampling methodology, with no significant association of concurrently obtained swab and biopsy samples from any individual. Interestingly, swabs appear less distally removed from environmental microbial communities than biopsy-derived samples in nmMDS, suggesting a closer community structure.

**Fig. 3:**
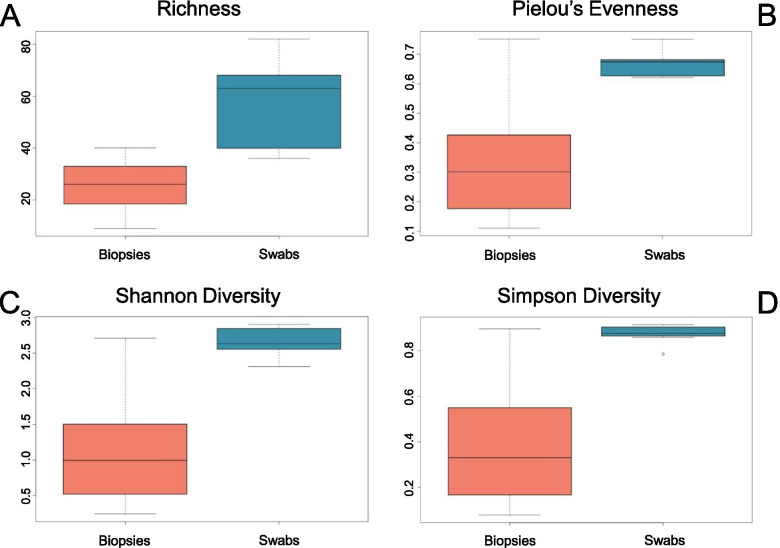
Alpha diversity indices of swab and biopsy results. Richness (**A**), evenness (**B**), Shannon (**C**) and inverted Simpson (**D**) diversity plots illustrate variation between biopsy (left) and swab (right) derived results. Calculations for diversity, evenness and richness were performed using in-built functions of R software, with box plots generated using the same program

Permutational multivariate analysis of variance (PERMANOVA) based on the standardised Bray-Curtis dissimilarity matrices and performed using Primer 7 supports significant influence of sampling methodology on composition of results (*p* = 0.01 and t = 2.689 with 979 unique permutations), with a notable but less significant difference between the two sampling timepoints represented within the dataset (*p* = 0.021) (Additional file 2). Use of a further PERMANOVA design based on Bray-Curtis to compare variation of grouped swab and biopsy samples across individual fish found no significant differences (Additional file 3). Unweighted UNIFRAC analysis provided similar results (Additional file 4). Alpha diversity indices of ASV richness, Pielou’s evenness, Shannon, and Simpson diversity from a rarefied dataset (Fig. 3) also illustrate a clear difference in community richness and various measures of diversity between sampling methodologies.

### Variation in isolated taxa with gill region sampled

When total identified ASVs were contrasted between swabs and corresponding gill biopsy samples, a subset of taxa were identified by both methodologies, but an overall greater number were not. A total of 73 ASVs were concurrently identified in both biopsy and swab-derived samples, whereas 45 were observed exclusively within the biopsy samples (but not in either swab or seawater samples), and a total of 230 ASVs were identified in swab-derived samples that were not seen in the biopsy samples. These findings are illustrated in Additional file 5, with supporting data in Additional files 6, 7, 8 and 9).

### Variation in microbial predominance across taxonomic levels with varied sampling methodology

Predominant taxa were determined as those composing an average abundance of 0.5% or more within a community, when surveyed at phylum (Additional file 10) and order (Additional file 11**)** level. The dominant phyla of swab-derived sampling were Proteobacteria (average abundance 72.8%), Bacteroidetes (22.3%), Chlamydiae (2.8%) and Verrucomicrobia (1.1%). The dominant phyla frequency of corresponding biopsy samples meanwhile were Proteobacteria (average abundance 87%), Bacteroidetes (5.7%), Chlamydiae (3.8%), Verrucomicrobia (0.7%), Firmicutes (0.7%) and Actinobacteria (0.5%). Although these abundances are relative within a compositional dataset, clear trends in dominant microbial taxa are seen. Relative abundance of Bacteroidetes is particularly variable, and was identified by applying Analysis of Composition of Microbiomes (ANCOM) [62] testing through the Qiime pipeline as significantly divergent across sampling methodologies. Varied relative abundance at order level between sampling methods was also detected using ANCOM testing for Flavobacteriales, Pseudomonadales Sphingomonadales and Rhodobacterales.

**Fig. 4:**
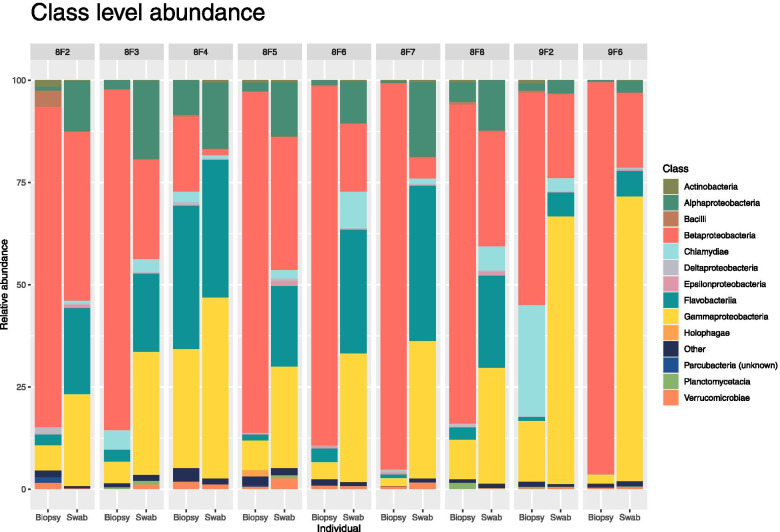
Stacked bar plot of Class level taxa. Stacked bar figure illustrating relative abundance of identified taxa at Class level across individual sampled fish. Variation in community composition of gill tissue obtained from the same individual using swab or biopsy methodology is visualised with paired stacked bars. Plots were generated using the ggplot package for R software

Comparison of swab and biopsy-derived microbial populations from specific individuals also demonstrated a clear difference in community composition using different sampling methodologies (Fig. 4). Intra-individual comparisons at class level allow observation of general trends in community composition, including a tendency towards dominance of Betaproteobacteria within biopsy-derived samples. Swab-derived results appear to contain a more even proportional abundance of observed taxa, with greater proportional composition of Flavobacteria, as well as Gamma- and Alphaproteobacteria (Fig. 4).

Differences in the relative compositional abundance of specific taxa were assessed using similarity percentage analysis (SIMPER) to determine the bacteria of greatest impact on observed variation between sampling methodologies (Table 1). A total of 17 genus-level ASV’s were identified to account for 49.98% of observed variance between swab and biopsy samples. The microbes identified as having the greatest contribution to this dissimilarity between groups were (in order); *Procabacteriaceae*; unclassified (8.23%), *Psychrobacter* (6.37%), Flavobacteriaceae; unclassified, (4.52%), *Candidatus* Piscichlamydia (2.93%), Rhodobacteraceae; unclassified (2.79%), *Loktanella sp* (2.79%), Chryseobacterium (2.68%) and *Candidatus* Branchiomonas (2.56%). Of these, *Procabacteriaceae*, *Candidatus* Branchiomonas and *Candidatus* Piscichlamydia demonstrate greater relative abundance within biopsy samples, and the remaining genera are generally of greater abundance in swab-derived samples (Table 1).

**Table 1:** Results of SIMPER analysis

Genus	Contrib%	Av.Diss	Diss/SD	Cum.%	Average proportional abundance biopsy	Average proportional abundance swabs
Procabacteriaceae; unclassified	8.23	5.66	1.72	8.23	69.8	19.5
Psychrobacter	6.37	4.38	2.09	14.6	1.7	14.2
Flavobacteriaceae; unclassified	4.52	3.11	1.82	19.12	2.8	10.2
Candidatus Piscichlamydia	2.93	2.02	1.47	22.05	3.8	2.7
Rhodobacteraceae; unclassified	2.79	1.92	2.21	24.84	0.3	3.2
Loktanella	2.79	1.92	1.8	27.63	0.2	3.1
Chryseobacterium	2.68	1.85	1.75	30.31	1.4	3.9
Candidatus Branchiomonas	2.56	1.76	0.88	32.87	4.3	1.3
Pseudoalteromonas	2.43	1.67	0.75	35.3	0.4	4.3
Sphingomonadaceae; unclassified	2.19	1.51	1.51	37.49	0.8	2.9
Flavobacterium	2.15	1.48	1.3	39.64	0.1	1.3
Photobacterium	2.05	1.41	0.62	41.69	0.1	1.1
Gelidibacter	2.04	1.41	2.03	43.73	0.3	1.6
Tenacibaculum	1.73	1.19	1.52	45.46	0.3	1.2
Shewanella	1.54	1.06	0.91	47	0.1	1.1
Vibrio	1.52	1.04	1	48.52	0.2	0.6
Aequorivita	1.46	1	1.92	49.98	0.1	0.9

Results of SIMPER analysis (performed using Primer 7) to determine the contribution of specific microbial taxa to overall dissimilarity between swabs and biopsy groups within square-root transformed Bray-Curtis resemblance dataset (Fig. 2). Average relative abundance (%) in both biopsy and swab datasets is presented alongside SIMPER analysis output

## Discussion

Resident microbial external epithelial communities of fish can be influenced by many factors, including environmental and host drivers of variation [63–65], and so every effort was made to minimise the impact of these factors on intra-individual methodological comparisons in this research. The influence of external variables in this research were considered to have been comparable within each fish at the time of sampling, with host factors such as physiological stress and nutritional status also having a consistent impact on gills [66, 67]. However, disease status and other contralateral impacts on community were identified as potential uncontrolled variables that might introduce intra-individual left-right variation in the microbial consortia of gills [12]. Every effort was therefore made to limit the impact of disease status on results. This was implemented by ensuring contralateral gills presented identically in gross assessment, and by excluding fish considered to be suffering anything more than mild gross gill pathology [68]. No active bacterial infections, including epitheliocystis, were observed through histological assessment of the gills in this study, although these assessments were conducted using second gill arches, and not the first arches obtained for sequencing. Samples for comparison of swabs and biopsies were obtained from contralateral gills as it was considered that swabbing a tissue prior to biopsy excision might disrupt the community subsequently collected. Identical anatomical regions of gills were, however, sampled from each first left and first right gill for swabbing and biopsies respectively (Fig. 1). As with any study of the microbiome, the influence of natural variation of microbiota across gill surfaces within an individual must be expected. However, results indicate consistent trends in communities obtained with varied sampling methodology, which is likely not explainable by natural geographical variation across the gill surface. Thus, the sampling methodology significantly impacts assessments of community richness and diversity (Fig. 3) as well as producing significant variation in the isolation of specific taxa at multiple taxonomic levels. Significant inter-individual variation of microbiota has been previously described in Atlantic salmon [69]. However, study of the microbiome in other species demonstrates fairly consistent intra-individual community structure when a consistent sampling methodology is employed across bilaterally symmetrical, physiologically comparable body sites, provided there is an absence of an on-going disease state and despite varied topographical sample collection [70, 71]. If a similar scenario applies then in fish, we might expect similarity of microbial consortia obtained from identical gill anatomical regions, despite inter-individual variation. Although the results of this research do identify notable variation in relative abundance of specific taxa within sampling methods (Additional files 10 and 11) sampling methodology does appear to be an important determinant factor in assessments of community composition, as evidenced by PERMANOVA analysis. Whilst individual variation was expected across left and right sampled gills, it was considered that the broad trends observed in bacterial richness and diversity of this research (Fig. 3) were unlikely to be due to a purely left/right sampling effect. Studies in the external epithelia and mucosal surfaces of humans find no significant left to right sampling differences unless clinical disease is present, although they do note subtle individual variation [72–74]. While a left right effect on these results cannot be excluded, disease is accounted for in this study by histopathology and gross scoring. Broad trends observed in bacterial richness and diversity of this research are therefore considered unlikely to be due to a purely left/right sampling effect, with sampling methodology the most likely driver of the variation in diversity observed.

Results therefore support a divergence in data obtained from identically treated swab and biopsy-derived high-throughput sequencing, indicating different sampling methodology likely impacts the microbial genomic material obtainable from Atlantic salmon. Significant variation in richness between biopsies and swabs was identified using a Mann-Whitney non-normal two group test (*P* = 0.0006). Although inference of statistical significance between diversity indices is not considered best practice [75–77] and so was not performed here, based on plotted results in Fig. 3, findings do suggest differences between the diversity of swab and biopsy sampling. Swabbing overall appears to collect a richer and more diverse microbiota, with apparent greater similarity to environmental isolate community composition (Fig. 2). Read counts obtained from swabbing were consistently higher both before and after filtration processing (Additional file 1). Biopsy-derived communities were of lower diversity and overall lower read counts in general. This trend is maintained across individuals, with varied microbial consortia obtained by altering sampling methodology and following a standard amplification and sequencing protocol. Biopsy-derived samples demonstrated more intra-individual variation overall, but communities were overall of lower richness and diversity.

An important influence on this study was the impact of host-DNA contamination in biopsy-derived samples. High presence of host genomic material can overwhelm low biomass microbial signals in sequencing reactions [78], resulting in artificially reduced community richness. Initial microbial biomass in DNA extractions is unknown for this study due to the sampling methodology employed. High biopsy mass relative to tissue mass on swabs is confounded by the greater presence of host tissue in biopsy samples. All but two of the swab-derived samples yielded greater read numbers before and after filtration when compared to biopsy samples. In addition, analysis of the filtration datasets demonstrated that our filtration removed an average of 11.5% of reads from swab-derived samples, compared to an average of 76.3% of total reads removed from biopsy-derived samples (Additional File 1). This is likely due to the negative impact of high levels of host-derived sequences within biopsy samples. Although rarefaction curves suggest sufficient sequencing depth was achieved (Additional file 1) [79], these indicators are only useful in assessment of the success in capturing available diversity within a sample, not ‘true’, original diversity that may have been excluded due to high contaminant DNA inclusion and resulting low microbial inclusion [80] within PCR reactions. Degree of inhibition in final sequencing results is therefore an important concurrent influence on observed variation between sampling methodologies in assessment of the salmonid microbiome, and an important consideration for study design. This impact is also demonstrated, for example, in the study of the human ileal pouch where, although mucosal-associated microbial communities that were isolated were compositionally similar, less invasive sampling yielded relatively more bacterial versus host DNA [81]. Our study was designed for identical treatment of samples except for collection methodology, however, it seems likely that sequencing of biopsy-derived samples from Atlantic salmon gills would have benefitted from a modified sequencing protocol with techniques for reduction of host contaminants. Biopsy samples overall yielded lower sequence reads, with a high proportion filtered as non-microbial (Additional file 1). To determine whether sub-surface and more cryptically located microbial communities of gill tissue are of lower biomass, further work might consider a equivolumetric comparison of different gill tissue locations [82]. For future studies that elect to utilise biopsies in analysis of the gill microbiome, use of PCR blockers, previously demonstrated as successful in marine diet studies [83, 84], or an altered protocol for low microbial biomass [85] would likely enhance the protocol.

Targeted molecular diagnostics for gill pathogens are often performed using tissue sections [86] with recent but as yet unpublished research exploring the use of gill clips from live fish to reduce the impact of sampling (through avoidance of euthanasia). However, swabbing is noted as having equivalence or even superiority in isolation of some pathogens, such as gill surface amoeba associated with amoebic gill disease (AGD) [87]. Swabbing is often the method of choice for sample collection in exploration of aquatic microbial ecology in other contexts, such as from catch-and-release of endangered wild fishes. This is likely due to it being relatively cheap and simple to perform, as well as being less invasive [88]. Although the current study represents the first published contrast of microbial sampling methodology of salmonids, many comparisons in human medical research assess the equivalence of swabs and biopsies in different medical scenarios, with varied outcomes. Guidance in sampling the microbiome of production species such as Atlantic salmon will be of great interest to fish health professionals, as recent research suggests important links of gill microbiota with altered fish health [89], as well as in mitigation of emerging disease [90]. Much research is conducted in the study of the gill microbiome to understand the influence of factors such as captive production facilities and how aging alters epithelial microbiota [91, 92], and so future research in methodology for sampling must include additional taxa, age classes and production systems to confirm the application of these findings across fish species and in varied contexts.

Overall, diversity and total read count results in this research seem to suggest that sampling the gill microbiota to answer broad ecological questions, such as for trends in microbial assemblage over time or across different populations, might best be answered using a swabbing methodology. Swabbing is non-destructive, with minimal tissue damage and relative repeatability [27] (except for any changes that might be introduced by removal of the gill mucus layers). Biopsies are considered the gold standard in many human and teleost microbial diagnostics, however, they suffer from PCR inhibition and lack of sequencing depth, which might hinder research when full communities are to be surveyed.

Our results mirror studies of the microbiota of human skin that found varied diversity of microbiota using different sampling methodologies, with this variation proposed to be due to the varied tissue depth sampled [42]. Clinical research in human health isolates significantly different microbial communities at varied healthy skin tissue sampling depth [81], suggesting a sub-dermal microbial community distinct from epithelial surface populations [42, 57]. Previous research in teleosts suggests that microbiota of the GIT also varies across different regions and with tissue depths [36, 37, 49, 50]. The potential may exist then for components of the gill microbiome to be selectively localised within cryptic tissue locations, such as beneath the surface epithelium. In other aquatic species, biopsies have been used successfully for assessment of deep tissue bacteria as part of infections, or proposed sub-surface symbionts [93]. Gills are composed predominantly of epithelial, goblet and ionocyte cell layers above supportive cartilage with an extensive vascular supply [94] and so lack a dermis, however, they do have a highly specialised tissue surface structure, with a large surface area that includes various regions unlikely to be entirely accessible to swabbing. The potential exists then for microbes that were isolated exclusively by biopsy excision to represent a community that cannot be accessed by swabbing.

A distinct community, if it exists, sampled by full-thickness tissue excision might reflect the microbiota of more ‘cryptic’ locations, possibly with lower aerobic demand, that can survive beneath the tissue surface or as part of hard-to-reach cartilaginous or intra-lamellar populations. Due to the complex ultrastructure of gills, it is problematic to quantify gill surface area sampled by swabbing and biopsies in this study. However, results replicate a realistic diversity survey of gills, where a single aspect might be sampled by swabbing, and biopsies must sample a more focused area, but with greater access to regions of the complex three-dimensional structure of gills. Swabbing likely isolated microbiota from the lateral gill surface, whilst biopsies samples more of the three-dimensional gill structure. Specific isolates identified only from biopsy sampling included those classified using the SILVA database as *Enterococcus*, *Peptoniphilus* and *Chryseobacterium*, all genera known to survive in anaerobic conditions (Additional file 7) [95]. Although taxonomic assignation based on sequencing regions of 16S fragments is error-prone due to similarity between related microbes, as well as due to the incomplete nature of the sequence databases, results identify the closest available taxonomic match for obtained sequences. These matches suggest that a number of the microbes identified exclusively by biopsy excision might be capable of anaerobic respiration and endospore formation. This trend might therefore suggest that bacterial isolates accessed by excision, which potentially reside in deeper tissue layers or more cryptic gill surface areas, vary in their properties from those with presumed greater environmental contact that are sampled by swabbing. Taxa identified as having the highest contribution to dissimilarity between results (Table 1) with greater average abundance in biopsy samples include *Candidatus* Piscichlamydia and *C*. Branchiomonas, both isolates known to be intracellular microbes of the gill epithelium and associated with the gill diseases epitheliocystis and complex gill disease [21, 96]. Interestingly, *Chryseobacterium* (of greater abundance in swabbing samples) and unclassified *Procabacteriaceae* (greater abundance in biopsy samples) are recognised intracellular bacteria of acanthamoeba [97]. There is currently no information available regarding the niche partitioning of variable microbial communities across the ultrastructure of gill tissue, but these results raise the interesting possibility of its existence. The greater similarity of swab-derived samples to environmental isolates (Fig. 2**,** Additional file 4) might indicate a closer association of swabbing-accessible communities to environmental populations, however this might also merely indicate greater incidental contamination of swab-derived isolates with environmental microbiota. Artificial similarity of community composition due to more diverse datasets derived from environmental and swab-derived samples, and lower diversity biopsy datasets, must also be considered. Overall, reduced diversity of biopsy samples presents an interesting novel question; might cryptic gill locations truly be of lower microbial diversity than surface mucus gill communities? Further work with optimised PCR conditions will assist in determining this. Regardless of this, our results demonstrate conclusively that sampling methodology impacts results.

## Conclusion

Overall, our results show a clear divergence in the microbial community composition that can be detected from fish when sampling method is altered. Use of varied methodology to collect microbiota, specifically biopsies and swabbing, impacts results. Although variation may be due in part to difficulties in processing biopsy-derived material, swabbing provided enhanced richness and diversity of gill microbiota relative to biopsies. Whilst our study details the gill microbiota of isogenic animals from a single location, results may also be applicable across important finfish species. Results therefore show that in addition to being a simple, non-destructive procedure, swabbing represents an effective methodology for collection of microbiota from fish as part of future aquaculture and ecological studies. Use of biopsies likely remains relevant though in the pursuit of specific research questions, including pathogen diagnostic studies, particularly for intracellular or more cryptically located microbes.

## Methods

### Sample collection

Atlantic salmon were sampled from a marine stage commercial farm by food incentivised crowd-netting from a single pen at the Scottish Sea Farms (SSF) Loch Spelve facility (56.374760, − 5.768232). The fish were 2 years old, of identical genetic background, and had been in sea water for 9 months at the first sampling, and 11 months at the second. Hand-netted fish were euthanised immediately by husbandry staff using immersion in 500 mg L^− 1^ 3-aminobenzoic acidethyl ester methanesulfonate (MS-222) to facilitate sample collection and prevent gill tissue disruption [98, 99]. This methodology was approved by the Animal Welfare and Ethics Committee at the University of St Andrews in line with European Union directive 2010/63UE. Brief post-mortem assessment of clinical gill pathology was performed for gross assessment of gills as per industry standard practice, with any macroscopic lesions noted before microbial samples were obtained using sterile technique. The samples for this study were collected as part of a larger histopathology study with multiple sampling time-points from the same population of fish. Fish for this study were obtained on samplings 8 and 9, which is reflected in the naming convention of samples. For example, 8F1 denotes Fish 1, from Sampling 8. Tissue was excised as biopsies from the left-side first gill arch gill and swabs were obtained from the right-side first gill arch for consistency. Tissue sections were approximately 1 cm wide, full length and full thickness biopsies and included both cartilage and lamellar gill tissue (Fig. 1). No effort was made to wash or dry tissue prior to placing in fixative in order to avoid disruption of the mucus layer and its associated microbiome. Swabs were obtained from a representative area on the contralateral gill (Fig. 1). Samples were identically fixed in 25 ml RNAlater solution (ThermoFisher Scientific). Fixed material was maintained at ambient temperature for approximately 24 h in RNAlater before long term cold storage at − 20 °C. Extractions were performed in duplicate for each fixed sample with a maximum of two freeze-thaw cycles to tissue. This provided biological replications of tissue biopsies and environmental controls, along with technical replicates from swabs.

### DNA extraction and quality control

DNA was extracted from fixed gill tissue using a modified protocol for DNeasy Blood and Tissue extraction kit (Qiagen). Tissue sections (10–12 mg) were mechanically disrupted using scissors prior to addition of DNeasy blood and tissue reagents [100]. Samples were air-dried in a laminar flow hood before use based on published advice for enhanced DNA collection from swabs [101] and to better facilitate removal of adherent material. Other protocols recommend blotting dry to remove excess RNAlater, but given the goals of this study, this was considered inappropriate for both swabs and tissue, and so air drying was instead employed. A sterile scalpel blade was used to scrape swab surfaces to collect adherent material. Extractions were then performed on both collect material and swab buds. Entire environmental samples were centrifuged at 3400 g for 10 min (Sigma 3–16 centrifuge with 11,180 rotor) and the majority of supernatant removed before being vortexed and transferred to microcentrifuge tubes. These were centrifuged at 15,600 g for 10 min (Eppendorf Centrifuge 5424), the supernatant removed and discarded, and the remaining liquid evaporated from the sample by air-drying in a laminar-flow hood. Samples were treated according to the manufacturer’s protocol of the DNeasy blood and tissue kit (Qiagen), modified to include agitation by vortexing at 15 min intervals for the first hour of incubation at 56 °C, followed by an overnight (12 h) incubation at 56 °C without agitation [102]. Following digestion, samples were briefly vortexed to ensure complete digestion and mixing of sample, before addition of 2 μl RNAase enzyme (Ambion) and gently mixed by inverting. A phenol-chloroform extraction step was incorporated into the protocol for both swabs and biopsies in response to initially high protein contamination in the biopsy samples [100]. High protein might have been mitigated by bleeding fish following euthanasia, however, this would have introduced a delay in sampling critically time sensitive gill tissue for microbial analysis and histopathology. Mass of biopsy tissue for extraction was minimised in line with kit recommendations to 10–12 mg, to limit protein inclusion. It was considered use of lower tissue biomass might negatively impact study results through limitation of gill surface area inclusion. Therefore, a phenol-chloroform step was introduced, rather than further reducing gill biopsy mass. Initial biomass of material from swabs is unknown, however, overall less host tissue was present in these samples. DNA extraction was then completed using the DNeasy Blood & Tissue kit (Qiagen) according to the manufacturer’s guidelines. Samples were eluted in 200 μl of buffer AE (Tris). DNA quality and quantity were analysed using a Nanodrop 1000 spectrophotometer. DNA concentration was measured, with repetition of the extraction of any sample less than 80 ng mL^− 1^. Purity and integrity of DNA was checked by measuring absorbance at 260:280 nm (> 1.8) and 230:260 nm (> 1.8) as well as observance of the DNA smear in 1% agarose gels with ethidium bromide. Duplicate DNA extractions were performed and DNA concentration rechecked for all extractions prior to pooling to a final concentration of 45 ng μl^− 1^ for sequencing. Average concentration of biopsy extractions was 56.1 ng / μl (standard deviation 24.5) and average DNA concentration of swab extractions was 30.3 ng / μl (standard deviation 13.4) prior to pooling.

### Next generation sequencing

Amplicon generation and library preparation for high-throughput sequencing was performed largely in accordance with the Illumina Metagenomic sequencing library preparation protocol (Illumina, 2013). Small modifications, detailed below, were made to optimise data yield and quality for the sample type. Primers 341f (5′-TCGTCGGCAGCGTCAGATGTGTATAAGAGACAGCCTACGGGNGGCWGCAG) and 805r.

(5′-GTCTCGTGGGCTCGGAGATGTGTATAAGAGACAGGACTACHVGGGTATCTAATCC) [103, 104] (TruSeq) were used for the amplification of the 16S rRNA gene V3-V4 region. Amplicon PCR’s were performed in triplicate for each sample as well as for controls (total = 78), using 25 ng of pooled template DNA and 0.5 units of KAPA HiFi HotStart ReadyMix (Roche) with 5 pmol of each primer in a total volume of 25 μl. Thermocycler conditions were 95 °C, 3 min, followed by 27 cycles at 95 °C for 30 s, 55 °C for 30 s and 72 °C for 30 s; final extension was 72 °C for 5 min, modified conditions based on manufacturers recommendations (Illumina). PCR products were purified using AMPure beads according to the manufacturer’s protocol, utilising 20 μl of AMPure XP beads per sample (Agencourt, Beckmann Coulter), and duplicate 200 μl washes in 80% ethanol before resuspension in 25 μl of 10 mM Tris pH 8.5 buffer to maximize genomic DNA yield and concentration. Quantified triplicate PCR products were normalised and pooled to a final DNA concentration of 1 ng μl^− 1^. Index PCR reactions were performed for attachment of Illumina sequencing adapters and dual indices (Nextera XT Index Kit, Illumina) using 5 μl of primer each from the Nextera XT kit’s A and D in a unique combination for each sample. Reactions were performed using 25 μl of 2x KAPA HiFi HotStart ReadyMix (Roche) and 15 ng total of pooled template DNA, instead of the recommended 5 μl sample and 10 μl of PCR-grade water, in a total reaction volume of 45 μl. Thermocycler conditions were 95 °C for 3 min, followed by 8 cycles at 95 °C for 30 s, 55 °C for 30 s and 72 °C for 30 s; final extension was 72 °C for 5 min. PCR products were cleaned using 56 μl AMPure XP beads (Agencourt, Beckmann Coulter) in accordance with the recommended protocol and eluted in 25 μl of 10 mM Tris pH 8.5 buffer according to the manufacturer’s protocol. DNA concentration of extractions, stocks and cleaned PCR reactions were obtained by use of the Qubit dsDNA BR Assay and Qubit dsDNA HS Assay kits (ThermoFisher Scientific) according to the manufacturer’s instructions with Qubit 4.0 Fluorometer (Invitrogen, ThermoFisher Scientific). Products from all samples were pooled in equimolar concentrations to a final library concentration of 4 nM. The resultant library was denatured and hybridised according to the manufacturer’s recommendations with a 20% PhiX spike-in. Pooled tagged amplicons were then sequenced using the 2 × 300 bp MiSeq reagent kit v3 (Illumina) according to the manufacturer’s protocol.

### 16S microbiome workflow

Demultiplexed next generation data from the sequencing of prepared libraries was denoised and filtered using open source DADA2 [105] within Qiime2 v2019.2 [106, 107] The following parameters were used for DADA2; trunc_len_f: 300; trunc_len_r: 279; trim_left_f: 27; trim_left_r: 15; max_ee: 2; trunc_q: 2; chimera_method: consensus; min_fold_parent_over_abundance: 1, to produce a table of amplicon sequence variants (ASVs) with total counts [39, 108]. Taxonomy was assigned to results using the SILVA 128 reference database (13.8 version) (Quast et al., 2013) with additional BlastN checking of high prevalence ASVs. Sequences assigned to chloroplasts, archaea, mitochondria and reads unassigned below kingdom level were removed for generation of the final dataset. Abundance profiles were calculated based on total read counts in individual samples for assessment of beta diversity. Alpha-diversity metrics were calculated from treatment medians of a rarefied dataset (1200 reads) based on Qiime data and plateau of sequencing depth. Only isolates that reached the rarefaction curve plateau were included. Resemblance matrices, beta diversity metrics and multivariate analysis were performed using the programs Primer version 7 and Permanova+. Additional figure generation and statistical testing was performed using Vegan and Bioconductor packages in R 3.5.0 [109]. Figures were generated using Primer version 7 and R. Additional statistical testing was performed using in-built features of R.

## Supplementary Information

**Additional file 1.** Rarefaction sequencing curves and total read counts. Rarefaction curves before (A) and following (B) filtration to remove taxonomic assignation out-with desired 16S microbial SILVA results. Clear plateaus are seen suggesting adequate sequencing depth was achieved at a depth of 1500 sequences from swab and biopsy samples. Accompanying table illustrates the total unfiltered read counts seen in curve A, as well as read counts as specific filtration steps towards final filtered read counts. Filtration steps were as follows 1) Removal of sequences unassigned to bacteria; 2) Removal of sequences assigned to archaea; 3) Removal of sequences assigned to mitochondria; 4) Removal of sequences assigned to chloroplasts; 5) Removal of sequences not taxonomically assigned below kingdom level.

**Additional file 2.** Results of PERMANOVA testing by sampling methodology. Square-root transformed Bray-Curtis similarity data was utilized for PERMANOVA analysis with fixed effect for mixed terms and 999 permutations. Standard error 2.395 (biopsy) and 2.1556 (swabs).

**Additional file 3.** Results of PERMANOVA testing by individual fish. Square-root transformed Bray-Curtis similarity data was utilized for PERMANOVA analysis with fixed effect for mixed terms and 999 permutations for individual fish (swabs and biopsies).

**Additional file 4.** UNIFRAC nmMDS. Filtered sequences were aligned using MAFFT [110], and phylogenetic tree built using FASTTREE [111] using default parameters within the QIIME2 pipeline. The resultant UNIFRAC distance matrix was used in generation of non-metric multidimensional scaling analysis as shown here. Results of PERMANOVA using unrestricted permutations for comparison of samples by sample type (swab;biopsy;environmental) shown in table indicate significant variation by sample type.

**Additional file 5.** Shared and unique taxa from different sampling methodologies. Euler (venn-type) diagram illustrates shared and uniquely identified taxa from specific sampling methodologies. This diagram was generated using the software eulerAPE.

**Additional file 6.** Taxa unique to biopsy sampling datasets. Uniquely identified ASV’s from biopsy sampling, including taxonomic assignation from SILVA database.

**Additional file 7.** Taxa unique to swabbing sampling datasets. Uniquely identified ASV’s from swab sampling, including taxonomic assignation from SILVA database.

**Additional file 8.** Taxa identified using both sampling methodologies. Shared ASV’s identified from both biopsy and swab sampling datasets, including taxonomic assignation from SILVA database.

**Additional file 9.** Summary table shared and unique taxa including environmental water data. Unique and shared ASV’s across entire dataset as accompaniment to Additional file 5.

**Additional file 10. **Phyla level box plot. Box plots illustrate the average community composition at phylum level obtained by swabbing (blue) and biopsy (pink) sampling methods. Significant (*P* < 0.01) differences in average relative abundance was detected using mann-whitney t-testing. Significant variation was detected between Bacteroidete results (*p* = 0.005).

**Additional file 11. **Order level box plot. Box plots illustrate the average community composition at order level obtained by swabbing (blue) and biopsy (pink) sampling methods. Significant (*P* < 0.01) differences in average relative abundance was detected using mann-whitney t-testing. At order level, significant variation was detected between swab and biopsy derived results for Flavobacteriales (0.005), Procabacteriales (0.001), Pseudomonadales (0.001), Sphingomonadales (0.005), Rhodobacterales (0.001), and Vibrionales (0.019).

## Data Availability

Data for this publication has been made available via the NCBI database under the associated accession number PRJNA667072.

## Abbreviations

AGD: Amoebic Gill Disease
ASV: Amplicon Sequence Variants
DNA: Deoxyribonucleic Acid
GIT: Gastrointestinal Tract
nmMDS: Non-metric Multi-Dimensional Scaling
PCR: Polymerase Chain Reaction
PERMANOVA: Permutational Multivariate Analysis of Variance
SSF: Scottish Sea Farms
SIMPER: Similarity Percentage analysis
