# Supplementary figures and images for: Sampling the fish gill microbiome: a comparison of tissue biopsies and swabs
Source: BMC Microbiol. 2021 Nov 10;21:313. doi: 10.1186/s12866-021-02374-0 (PMC8579561; doi:10.1186/s12866-021-02374-0)

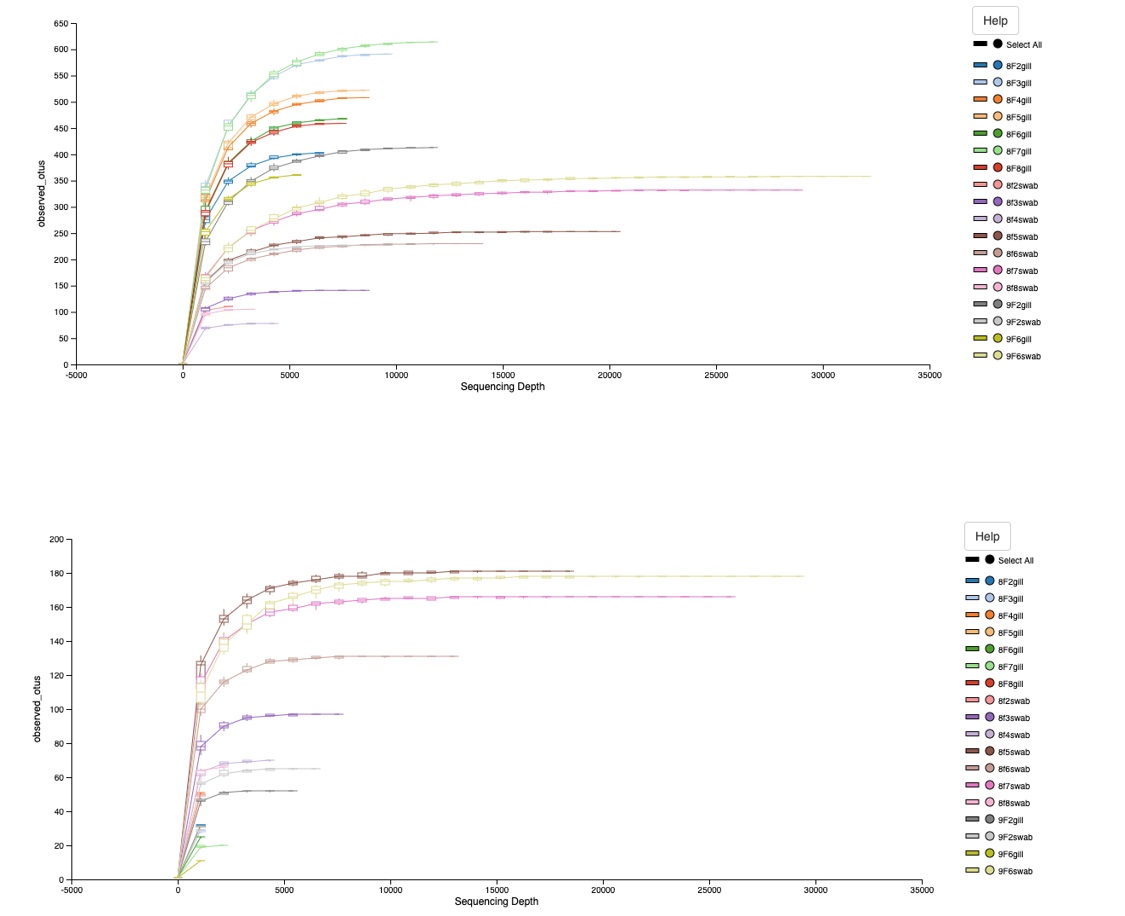

Supplement: Supplementary file 1 — Additional file 1. Rarefaction sequencing curves and total read counts. Rarefaction curves before (A) and following (B) filtration to remove taxonomic assignation out-with desired 16S microbial SILVA results. Clear plateaus are seen suggesting adequate sequencing depth was achieved at a depth of 1500 sequences from swab and biopsy samples. Accompanying table illustrates the total unfiltered read counts seen in curve A, as well as read counts as specific filtration steps towards final filtered read counts. Filtration steps were as follows 1) Removal of sequences unassigned to bacteria; 2) Removal of sequences assigned to archaea; 3) Removal of sequences assigned to mitochondria; 4) Removal of sequences assigned to chloroplasts; 5) Removal of sequences not taxonomically assigned below kingdom level. [file 12866_2021_2374_MOESM1_ESM.jpg]

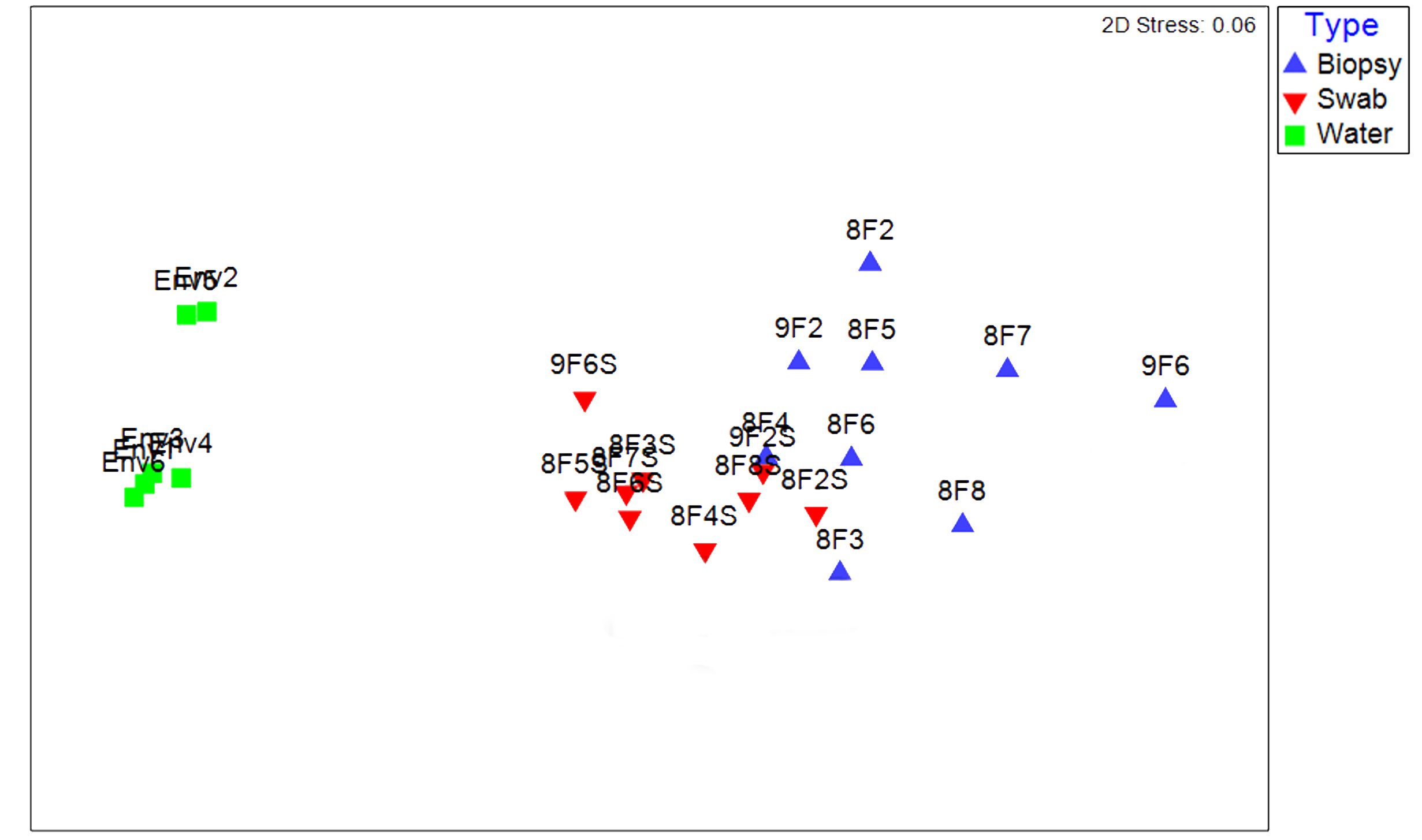

Supplement: Supplementary file 4 — Additional file 4. UNIFRAC nmMDS. Filtered sequences were aligned using MAFFT [110], and phylogenetic tree built using FASTTREE [111] using default parameters within the QIIME2 pipeline. The resultant UNIFRAC distance matrix was used in generation of non-metric multidimensional scaling analysis as shown here. Results of PERMANOVA using unrestricted permutations for comparison of samples by sample type (swab;biopsy;environmental) shown in table indicate significant variation by sample type. [file 12866_2021_2374_MOESM4_ESM.zip › Additional file 4 nmMDS unifrac.png]

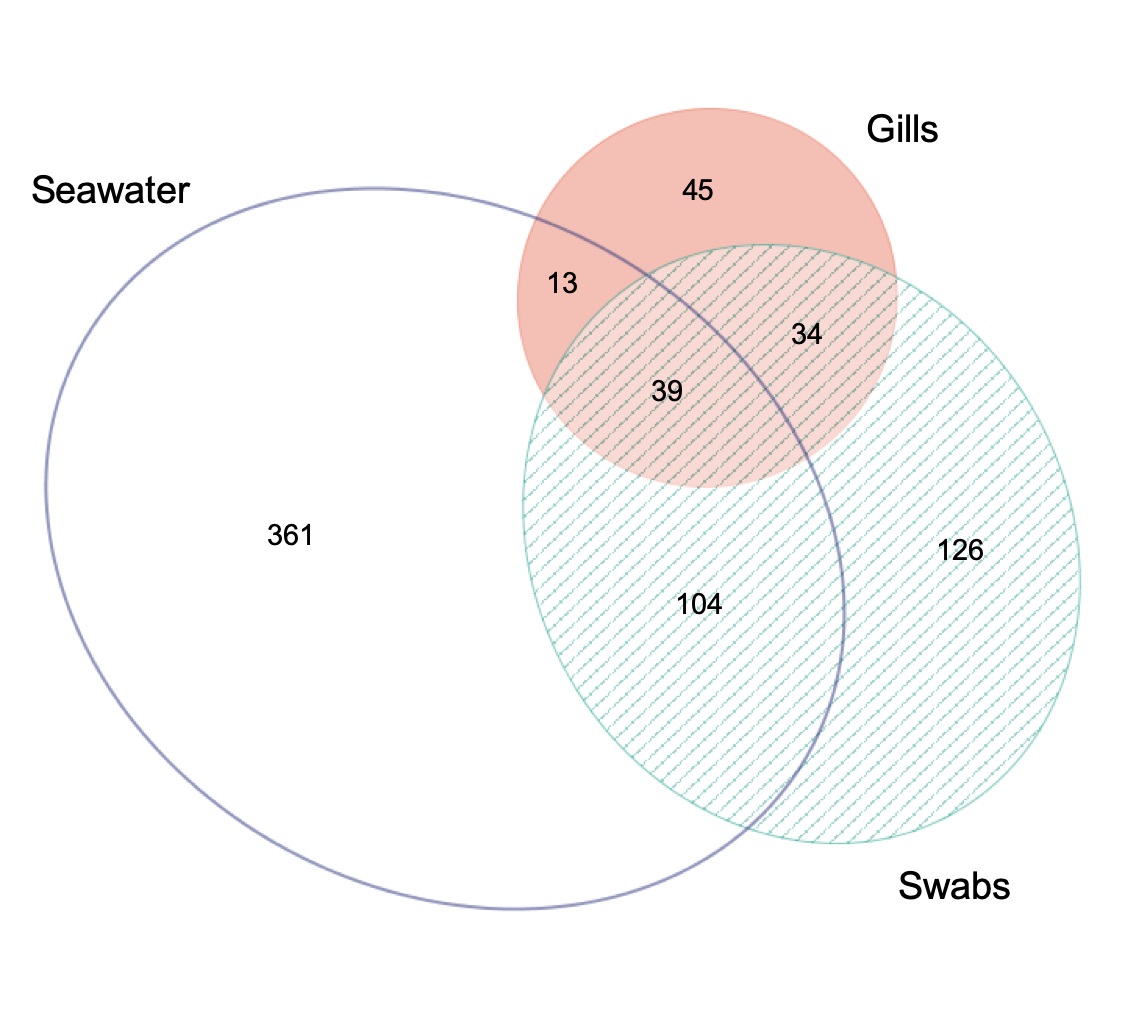

Supplement: Supplementary file 5 — Additional file 5. Shared and unique taxa from different sampling methodologies. Euler (venn-type) diagram illustrates shared and uniquely identified taxa from specific sampling methodologies. This diagram was generated using the software eulerAPE. [file 12866_2021_2374_MOESM5_ESM.jpg]

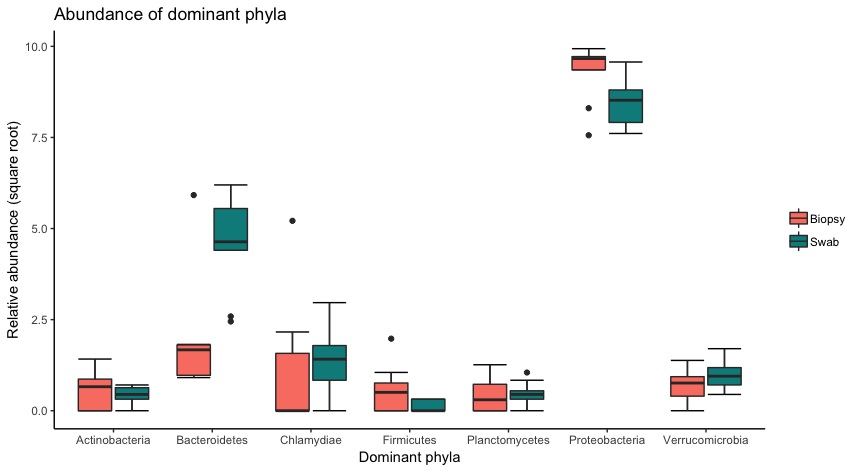

Supplement: Supplementary file 10 — Additional file 10. Phyla level box plot. Box plots illustrate the average community composition at phylum level obtained by swabbing (blue) and biopsy (pink) sampling methods. Significant (P < 0.01) differences in average relative abundance was detected using mann-whitney t-testing. Significant variation was detected between Bacteroidete results (p = 0.005). [file 12866_2021_2374_MOESM10_ESM.jpg]

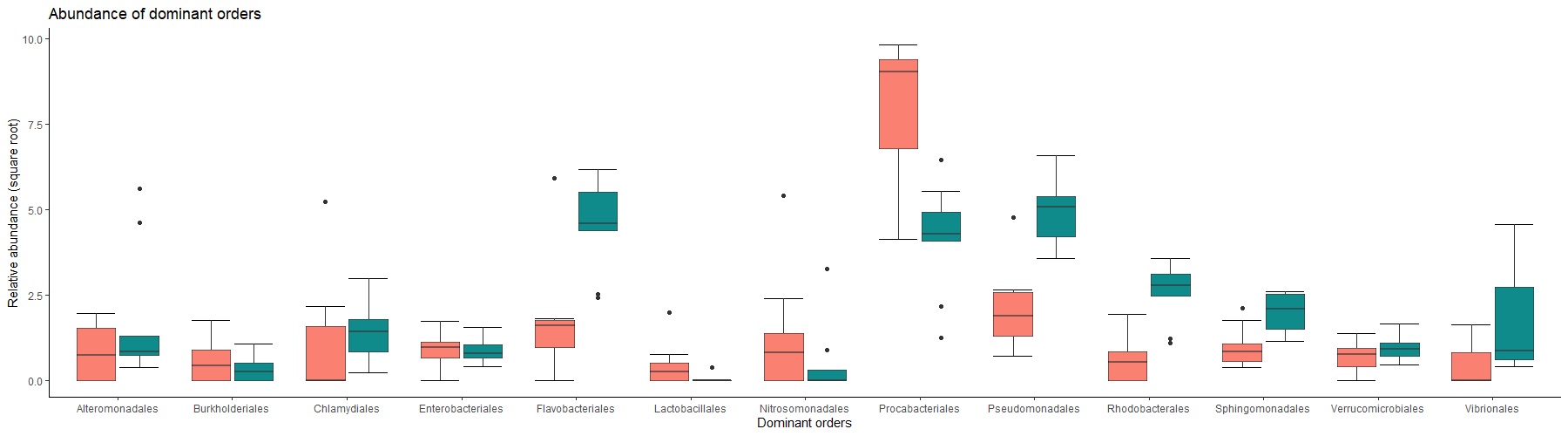

Supplement: Supplementary file 11 — Additional file 11. Order level box plot. Box plots illustrate the average community composition at order level obtained by swabbing (blue) and biopsy (pink) sampling methods. Significant (P < 0.01) differences in average relative abundance was detected using mann-whitney t-testing. At order level, significant variation was detected between swab and biopsy derived results for Flavobacteriales (0.005), Procabacteriales (0.001), Pseudomonadales (0.001), Sphingomonadales (0.005), Rhodobacterales (0.001), and Vibrionales (0.019). [file 12866_2021_2374_MOESM11_ESM.jpg]
